# Legacy of Tehran Lipid and Glucose Study; Evidence on Reproductive Lifespan and Cardiometabolic Health

**DOI:** 10.5812/ijem-167142

**Published:** 2026-01-31

**Authors:** Maryam Farahmand, Maryam Mousavi, Marzieh Saei Ghare Naz, Mahbanoo Farhadi-Azar, Mahsa Noroozzadeh, Fereidoun Azizi, Fahimeh Ramezani Tehrani

**Affiliations:** 1 Reproductive Endocrinology Research Center, Research Institute for Endocrine Molecular Biology, Research Institute for Endocrine Sciences, Shahid Beheshti University of Medical Sciences, Tehran, Iran; 2 Endocrine Research Center, Research Institute for Endocrine Disorders, Research Institute for Endocrine Sciences, Shahid Beheshti University of Medical Sciences, Tehran, Iran; 3 Foundation for Research & Education Excellence, Vestavia Hills, Al, USA

**Keywords:** Menarche, Menopause, Endogenous Estrogen Exposure, Lactation, Hormonal Use, Tehran Lipid and Glucose Study (TLGS)

## Abstract

**Context:**

Understanding the different reproductive factors and their link to non-communicable diseases (NCDs) is crucial in a community-based cohort study. The present study provides a comprehensive synthesis of published findings derived from the Tehran lipid and glucose study (TLGS) pertaining to women, offering insights into the epidemiological patterns of reproductive lifespan and its impact on cardiometabolic parameters.

**Evidence Acquisition:**

We conducted a thorough review of all studies on reproductive lifespan conducted within the TLGS framework.

**Results:**

Overall, the mean (SD) age at menarche among participants was 13.35 (1.5) years, whereas the mean (SD) age at menopause was 50.16 (5.7) years. Early menarche was identified as a significant factor associated with later metabolic impairments, including higher odds of prediabetes (OR: 2.7; 95% CI: 1.1 - 6.6), type 2 diabetes mellitus (DM) (OR: 3.6; 95% CI: 1.2 - 10.7), and metabolic syndrome (MetS) (OR: 2.3; 95% CI: 1.1 - 5.4) in fully adjusted models. Comparisons between surgical and natural menopause revealed that the incidence of MetS was nearly tenfold higher among women who experienced surgical menopause, accompanied by elevated mean fasting plasma glucose (FPG) and 2‑hour post‑load glucose concentrations. Furthermore, each additional year in age at natural menopause was associated with a 10% increase in the incidence of chronic kidney disease (CKD), whereas prolonged exposure to endogenous estrogen appeared to confer protective effects, reducing the incidence of hypertension, cardiovascular disease (CVD), CKD, and osteoporotic fractures. In addition, lactation was found to markedly lower the risk of MetS among women with a prior history of gestational diabetes, emphasizing its potential long‑term cardiometabolic benefits.

**Conclusions:**

The reproductive milestones significantly shape women’s long‑term cardiometabolic health, supporting the integration of reproductive history into preventive care models and longitudinal risk assessment frameworks.

## 1. Context

Currently, few cohort studies provide population-based evidence on reproductive histories throughout a person's reproductive years (1). This gap highlights a significant lack of understanding about reproductive health. Most information about reproductive issues and their related effects has come mainly from clinical settings or case-control studies (2). Unfortunately, these study types often face limitations such as selection bias, small sample sizes, and a lack of long-term data, which can reduce the reliability and usefulness of their findings. Therefore, a more comprehensive approach is necessary to evaluate and address reproductive health concerns over time effectively (1, 2).

Data collected on reproductive domains in Tehran, especially from the Tehran lipid and glucose study (TLGS), provide valuable insights into the reproductive patterns of an urban population in West Asia. Using reliable assessment tools, including structured questionnaires, thorough physical examinations, detailed reviews of medical histories and hospital records, as well as comprehensive biochemical and hormonal evaluations, this data set functions as an essential resource for filling knowledge gaps within a population-based cohort. This paper covers various topics, such as the age at menarche, its trends, influencing factors, and related cardiometabolic diseases; menopause and its influencing factors; disease consequences following menopause; prediction of menopausal age; lactation and women's hormonal use; and introduces endogenous estrogen exposure (EEE) duration as a variable involving reproductive factors, and its relationship with non-communicable diseases (NCDs).

## 2. Evidence Acquisition

In the TLGS, a detailed questionnaire focusing on reproductive lifespan was administered through face-to-face interviews conducted by trained personnel. This questionnaire covered a range of topics, including menarche, menopause, menstrual regularity, and pregnancy complications, which were assessed based on self-reported data under standard definitions for each complication. It also addressed abortion, types and durations of contraception use, and lactation. Participants' blood samples were collected at each visit. Serum concentrations of various biochemical parameters were measured promptly, while the remaining sera were stored at -80°C for future analysis (3). In this review, we focused solely on studies related to reproductive lifespan within the context of the TLGS.

## 3. Results

### 3.1. Menarche

Menarche, the onset of first menstruation, represents a pivotal milestone in female pubertal development and marks a key event in the maturation of the reproductive system (4). To determine the menarcheal age (MA), data from all phases of the TLGS were compiled, with MA defined according to the first self‑reported value as well as the most frequently reported age across assessments. During data collection, participants were asked: “At what age did you experience your first menstrual period?” An MA of ≤ 11 years was classified as early menarche, an MA of ≥ 16 years as late menarche, and an MA between 12 and 15 years as within the normal range. Based on these definitions, the distribution of early, normal, and late menarche in the cohort was 7.1%, 85.3%, and 7.6%, respectively.

#### 3.1.1. Menarche and Its Influencing Factors

The timing of menarche is determined by a complex interplay of genetic and environmental factors, including ethnicity, socioeconomic status, geographical location, nutritional status, physical activity level, Body Mass Index (BMI), central adiposity, overall body fat composition, and psychosocial influences (5-8). In nutritional status, TLGS results demonstrated that greater milk consumption [OR: 2.28; 95% CI: 1.03 - 5.05], calcium [OR: 3.20; 95% CI: 1.39 - 7.42], magnesium [OR: 2.43; 95% CI: 1.12 - 5.27], and phosphorus [OR: 3.37; 95% CI: 1.44 - 7.87] can lead to early menarche after adjusting for energy and protein intake and maternal MA (3). No significant association was observed between MA and lipid profile parameters; however, MA demonstrated significant correlations with height, BMI, waist circumference, and maternal educational level (4).

#### 3.1.2. Trend of Age at Menarche

The TLGS comprises seven follow‑up phases conducted at approximately three‑year intervals. MA was first assessed during the second phase using a self‑reported questionnaire and subsequently reassessed in each follow‑up phase. The mean (SD) MA among TLGS participants was 13.35 (1.5) years. There was a negative secular trend, with a reduction in MA (9) from 13.8 years to 12.9 years in women born between 1930 and 1990. A positive secular trend in mean height accompanied the declining trend in MA. Specifically, for each decade, the average MA decreased by 0.15 years, while the mean height increased by 0.99 cm (6).

#### 3.1.3. The Association Between Changes in Anthropometric Indices and Metabolic Syndrome with MA Across Various Birth Cohorts

**Figure 1.**
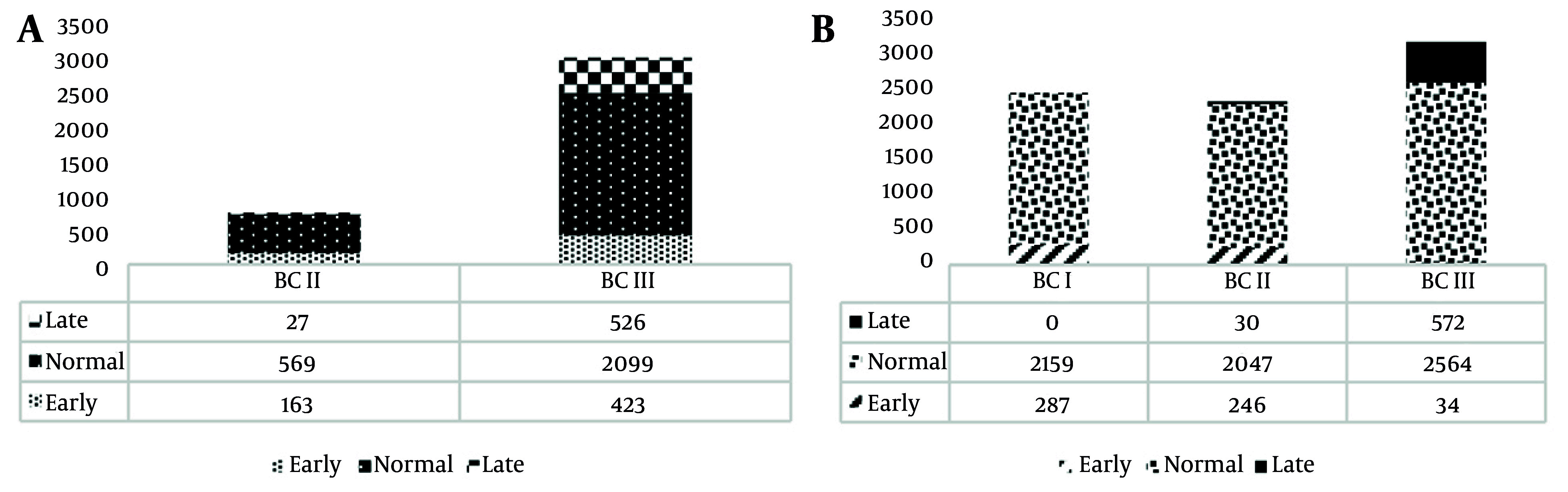
Distribution of menarche and menopause age groups among Tehran lipid and glucose study (TLGS) participants across various BC. Note: BC, birth cohort. BCI, ≥ 1980; BCII, 1960 - 1979; BCIII, ≤ 1959. Menarche age 15, late; 12 ≤ menarche age ≤ 15, normal. Menopausal age 55, late; 45 ≤ menopausal age ≤ 55, normal.

Figure 1A illustrates the distribution of MA categories across different birth cohorts (BCs). Within the TLGS framework, the findings indicated that the inverse association between MA and anthropometric measures — including mean BMI, waist‑to‑height ratio, and waist circumference — was more pronounced among older women compared with younger age groups (10). Another study stratified women from different BCs into three groups and found, in the adjusted model, that early menarche was associated with a 34% increased risk of MetS (95% CI: 1.04 - 1.71). Notably, within the early menarche group, the prevalence of MetS was higher among participants in the first two BC groups compared with the third (11).

#### 3.1.4. The Relationship of Menarcheal Age and Non-communicable Diseases

**Figure 2.**
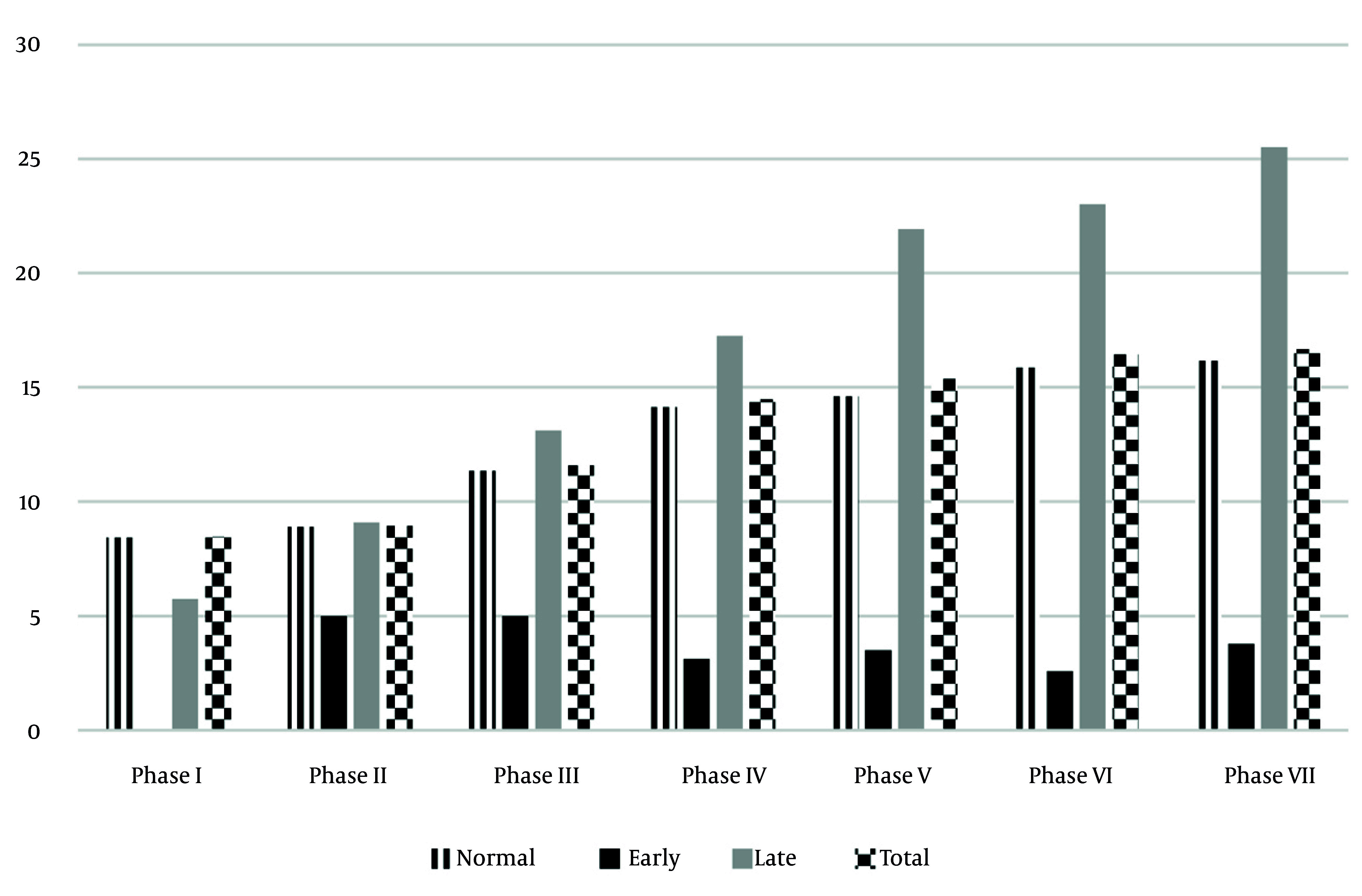
Prevalence of cardiovascular disease (CVD) by menarcheal age (MA) in Tehran lipid and glucose study (TLGS) participants across different phases. Note: Menarche age 15 is late; and between 12 and 15 is normal

Menarcheal age constitutes a fundamental indicator of female reproductive health. Within the TLGS, multiple investigations have evaluated the associations between MA and NCDs. For the assessment of cardiovascular disease (CVD) prevalence by MA category, analyses included women participating in various TLGS phases, specifically those aged over 40 years (Figure 2). Early menarche was independently associated with an increased risk of prediabetes (OR: 2.7; 95% CI: 1.1 - 6.6) and type 2 diabetes mellitus (DM) (OR: 3.6; 95% CI: 1.2 - 10.7) after adjustment for confounding variables (12). Furthermore, compared to the reference group (13 - 14 years), early MA conferred a greater risk of MetS (OR: 2.3, 95% CI: 1.1 - 5.4) and its individual components, including central obesity [OR: 2.5, 95% CI: 1.5 - 4.2], elevated blood pressure [OR: 2.9, 95% CI: 1.4 - 6.0], and higher fasting plasma glucose (FPG) levels [OR: 3.0, 95% CI: 1.4 - 6.0] (13). Employing the World Health Organization (WHO) standardized population, the prevalence of MetS was estimated at 11.5% (95% CI: 10.7 - 12.5), while in the Tehran population it was 11.7% (95% CI: 10.7 - 12.6). A 2023 longitudinal study further investigated the relationship between MA and the incidence of hypertension, revealing that women with late menarche displayed a 2.04-fold increased risk of developing arterial hypertension when compared to those whose MA fell within the normative range (12 - 15 years) (14). Additionally, earlier menarche has been shown to increase the likelihood of menstrual irregularity among reproductive-aged women (15).

### 3.2. Menopause

Menopause represents a critical life stage for women, signifying both the cessation of reproductive capacity and a potential increase in the risk of metabolic disorders. Owing to the significance of menopause in women's health, numerous investigations have been conducted within the framework of the TLGS. According to the WHO, natural menopause is defined as the permanent cessation of menstruation, confirmed by the absence of spontaneous menses for at least 12 consecutive months when no other pathological or physiological cause is identified (14). The age at menopause (AAM) is categorized as follows: Early menopause (AAM < 45 years), late menopause (AAM > 55 years), and normal menopause (AAM 45 - 55 years). Analysis of TLGS participants revealed the distribution of menopausal age categories as follows: 15.5% experienced early menopause, 70% had menopause in the normal age range, and 14.5% underwent late menopause.

#### 3.2.1. Age at Menopause and Its Influencing Factors

In participants of the TLGS, the mean age at natural menopause (ANM) was 50.16 years, with a standard deviation of 5.7 years. Besides genetics, smoking status, parity, history of chronic disorders, and MA were found to significantly influence the timing of natural menopause (16-20). Additionally, a history of chronic conditions, including DM [HR: 1.75; 95% CI: 1.32 - 2.32], hypertension [HR: 2.11; 95% CI: 1.64 - 2.72], MetS [HR: 2.01; 95% CI: 1.57 - 2.58], CVD [HR: 3.02; 95% CI: 1.93 - 4.73], chronic kidney disease (CKD) [HR: 2.64; 95% CI: 2.16 - 3.22], and thyroid disorders [HR: 1.41; 95% CI: 1.06 - 1.88], was associated with an increased risk of earlier onset of menopause. In contrast, dyslipidemia did not exhibit a significant association with the ANM (21).

#### 3.2.2. Trend of Age at Menopause

In total, the distribution of menopausal age categories across different BCs is shown in Figure 1B. In this regard, a study on women who participated in TLGS and were born between 1930 and 1960 was conducted, revealing that the average AAM for women born in the 1930s was 48.5, for those born in the 1940s was 49.5, and for those born in the 1950s was 49.9 years. After adjusting for potential confounders, the upward trend in menopausal age remained significant (P = 0.029) (22).

#### 3.2.3. Prediction of Age at Natural Menopause

Availability of frozen baseline serum of TLGS participants enabled us to precisely predict the ANM according to their serum concentration of anti-Mullerian hormone (AMH) (23-26). Anti-Mullerian hormone, which reflects the ovarian follicle pool size, serves as a biomarker predictive of menopause onset (27-29). The median difference between actual and predicted ANM was 0.51 years, as assessed by the Bland-Altman method, demonstrating good concordance between observed and predicted values in our setting (30). Notably, incorporating multiple AMH measurements over time has been shown to improve the precision of ANM predictions (31-33).

Given that both early and late menopause are linked to increased risks of specific conditions, including certain cancers and cardiometabolic disorders, women with diminished ovarian reserve, indicated by lower AMH levels, may be predisposed to CVD risk factors even during their reproductive years (34). The association between long-term exposure to ambient air pollutants and serum AMH concentrations, as well as the rate of AMH decline, in TLGS participants was investigated (35). Compared to women in the lowest tertile of exposure, no statistically significant differences were observed in the AMH decline rate for those in the second or third tertiles of air pollutant exposure. However, a possible link between elevated Cu levels and diminished AMH concentrations was observed (35). This aligns with several recent epidemiological studies reporting limited or inconsistent associations between ambient air pollution and AMH levels or ovarian reserve markers.

Additionally, another study on TLGS participants shows that dietary intake of dairy products appears to elevate serum AMH concentrations in women with regular menstrual cycles, suggesting a potential modifiable factor influencing ovarian reserve markers (31, 36).

#### 3.2.4. Menopause and Anthropometric Indices

The long-term effects of menopause on changes in adiposity indices were investigated using longitudinal data from 3,876 premenopausal women aged over 20 years, who were followed from 1998 to 2018. Participants were stratified into two groups: Those who transitioned to menopause during the follow-up period and those who remained premenopausal. At the conclusion of the study, 41.8% of the cohort remained in their reproductive phase. Compared to women who did not experience menopause, those who transitioned to menopause exhibited a 5% reduction in the odds of general obesity (OR: 0.95; 95% CI: 0.90 - 0.99) but a 6% increase in the odds of central obesity (OR: 1.06; 95% CI: 1.01 - 1.12). Among the assessed anthropometric indices, BMI demonstrated a positive association with ANM, whereas Body Shape Index and lipid accumulation product were inversely correlated with ANM, suggesting complex differential relationships between regional adiposity and timing of menopause (37).

These findings align with broader evidence indicating that menopause is accompanied by significant alterations in body composition, including increased central adiposity and concomitant shifts in metabolic risk profiles (38-41). Estrogen decline during menopause is known to promote fat redistribution favoring visceral accumulation, thereby elevating cardiometabolic risk despite a possible reduction in overall obesity prevalence (42). Furthermore, changes in lean mass and alterations in adipokine profiles contribute to these adverse body composition transitions observed during and after menopausal transition (5).

#### 3.2.5. The Relationship of Menopause and Non-communicable Diseases, Considering the Type of Menopause

**Figure 3.**
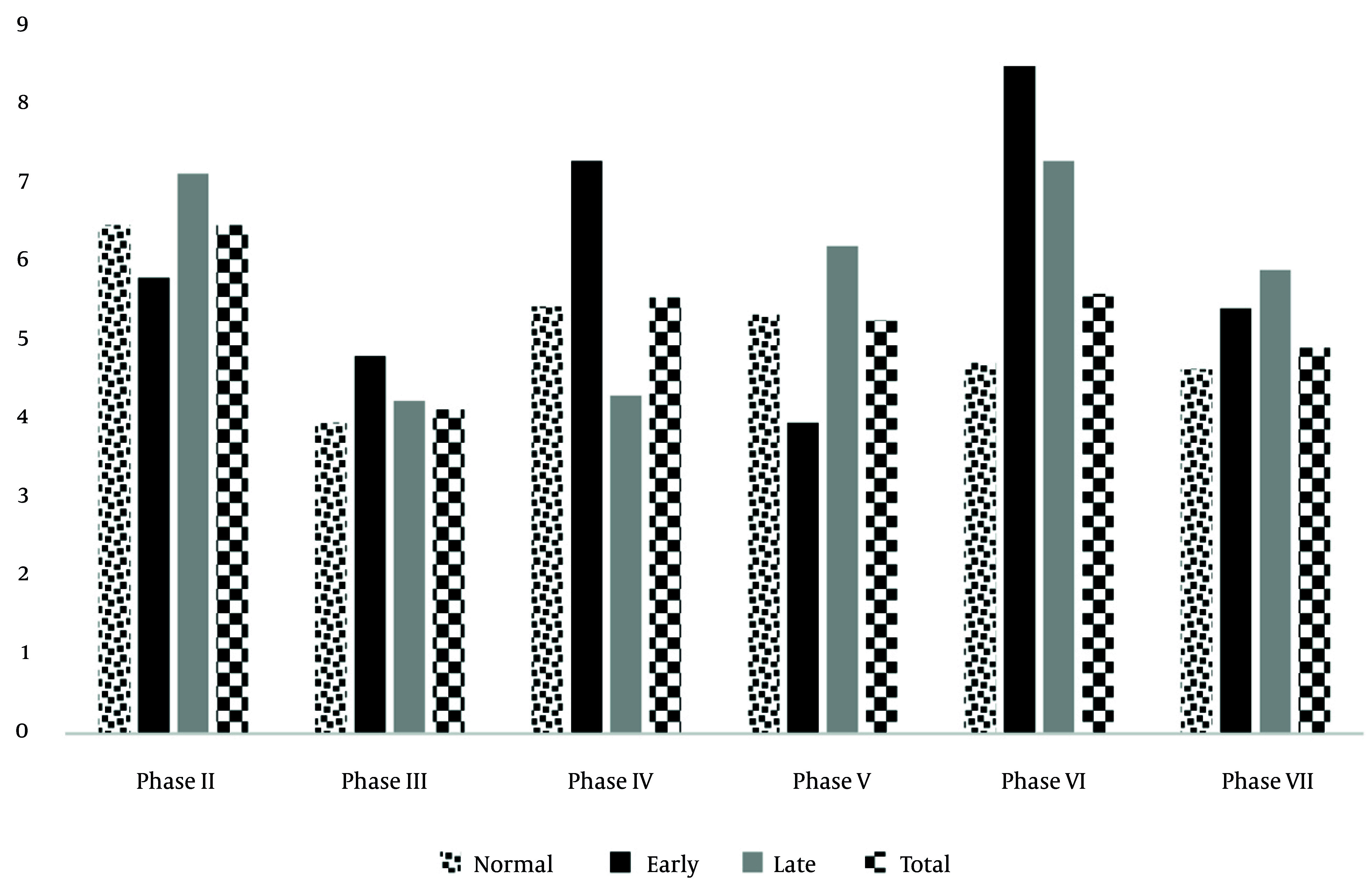
Incidence of type 2 diabetes by menopausal age in Tehran lipid and glucose study (TLGS) participants across different phases. Note: Menopause age 55 is late; 45 ≤ menopause age ≤ 55 is normal

The ovaries constitute a multifaceted endocrine organ that plays a central role in numerous metabolic processes beyond their reproductive functions (43). In the TLGS, the incidence of DM among menopausal women aged over 20 years, monitored across multiple TLGS phases, is presented in Figure 3. Menopause can occur naturally as a gradual decline in ovarian hormone synthesis or abruptly due to surgical intervention. Notably, the incidence of MetS was observed to be nearly tenfold higher in women undergoing surgical menopause compared to those experiencing natural menopause within three years before and after the menopausal transition (44). Surgical menopausal women also exhibited significantly elevated mean FPG and two-hour plasma glucose levels relative to their counterparts with natural menopause (45). Further analysis comparing cardiometabolic outcomes between women subjected to bilateral or unilateral oophorectomy — both in combination with hysterectomy — and those undergoing natural menopause revealed the highest odds ratio (OR) for MetS (OR = 5.0) in women with bilateral oophorectomy with hysterectomy. Moreover, women experiencing unilateral oophorectomy with hysterectomy demonstrated higher mean FPG levels compared to natural menopausal women, underscoring the exacerbated metabolic risk conferred by ovarian removal (46).

In addition, the timing of menopause exerts an influence on the incidence of CKD post-menopause. Evidence indicates that each one-year increase in ANM is associated with a 10% higher incidence of CKD, highlighting the complex interplay between reproductive aging and renal health outcomes (15).

These findings collectively emphasize the ovaries’ integral role in metabolic regulation and the profound systemic consequences of ovarian hormone depletion, particularly following surgical menopause, warranting targeted clinical attention during the menopausal transition.

### 3.3. Endogenous Estrogen Exposure

Various reproductive factors significantly influence the total duration of EEE in women. These factors include MA, the number and cumulative length of pregnancies, duration of breastfeeding (BF), usage of oral contraceptives, and AAM. Collectively, these variables determine the length of a woman's reproductive years during which endogenous estrogen levels fluctuate and exert physiological effects. For example, earlier menarche and later menopause extend the reproductive span, thereby increasing cumulative estrogen exposure, while pregnancies and lactation periods temporarily reduce exposure due to hormonal changes. Furthermore, oral contraceptive use may modulate endogenous estrogen levels depending on formulation and duration of use. Quantifying the combined impact of these reproductive factors is crucial, as longer EEE has been associated with modulating risks of various chronic conditions, including CVD and CKD (47, 48).

#### 3.3.1. Endogenous Estrogen Exposure Duration and Non-communicable Diseases

Community-based cohort studies analogous to the TLGS provide a valuable framework for evaluating the impact of EEE duration on NCDs in women. Several investigations have assessed this association by quantifying EEE as the interval between menarche and menopause, adjusted for factors such as pregnancies and lactation.

In one large study encompassing all female participants, including both reproductive-age and menopausal women, subjects were stratified into tertiles of EEE duration, namely T1 (lowest), T2, and T3 (highest) exposure groups. The incidence of CVD was 10.9 per 1000 person-years (95% CI, 9.4 - 12.8) in the lowest tertile, decreasing progressively to 7.2 (CI, 6.0 - 8.7) and 5.1 (CI, 4.1 - 6.4) per 1000 person-years in the middle and highest tertiles, respectively. These findings demonstrate a significant inverse dose-response relationship between EEE duration and CVD incidence, indicating longer EEE confers cardiovascular protection (47).

Another study conducted within the TLGS cohort categorized participants into two groups based on EEE duration (< 11 years versus ≥ 11 years) and examined CKD incidence. The overall cumulative incidence rate of CKD was 50.1 per 1000 person-years (95% CI: 47.7 - 52.6), with rates higher among women with shorter EEE (< 11 years) at 53.9 (95% CI: 50.2 - 57.8) compared to 47.1 (95% CI: 44.0 - 50.4) per 1000 person-years in those with longer EEE (≥ 11 years) (48). This suggests that extended exposure to endogenous estrogen may also confer renal protective effects.

Collectively, these studies underscore the importance of reproductive lifespan and cumulative estrogen exposure as critical determinants of cardiovascular and renal health in women, supporting the hypothesis that estrogen exerts protective effects against the development of multiple chronic diseases. The effects of EEE duration on the incidence of fractures and hypertension, two major NCDs, have been systematically evaluated in the TLGS. To assess the impact of EEE on fracture risk, all eligible postmenarcheal women, including 2,411 premenopausal and 2,858 menopausal participants, were included. After adjustment for potential confounders, the EEE z-score was found to be inversely associated with fracture incidence (adjusted HR: 0.70; 95% CI: 0.58 - 0.86), indicating that a shorter duration of EEE significantly increases fracture risk (49). This finding is consistent with evidence supporting the protective role of estrogen in maintaining bone density and reducing fracture susceptibility.

Additionally, the effect of EEE duration on hypertension incidence was examined in a cohort of 4,463 normotensive postmenarcheal women, comprising 3,599 premenopausal and 864 menopausal women, followed over a median period of 33.2 years (interquartile range 25.1 - 42.3). Following adjustment for confounders, results demonstrated that a longer EEE duration was significantly associated with a decreased incidence of hypertension, emphasizing the cardiovascular protective effects of prolonged EEE during a woman’s reproductive lifespan (50).

#### 3.3.2. Endogenous Estrogen Exposure and Body Composition

In a cross-sectional study conducted within the TLGS framework, 960 postmenopausal women aged over 40 years were assessed to investigate the association between EEE duration and body composition indices. For each additional year of EEE, fat mass decreased by 0.12 kg, skeletal muscle mass by 0.04 kg, fat-free mass by 0.07 kg, and the fat mass ratio by 0.003 (51). These results suggest that prolonged exposure to endogenous estrogen is associated with favorable changes in body composition, including reduced adiposity and alterations in muscle mass, which may have important implications for cardiometabolic health in postmenopausal women.

### 3.4. Lactation

BF promotes metabolic benefits in lactating mothers by enhancing energy expenditure, facilitating postpartum weight loss, increasing high-density lipoprotein cholesterol (HDL-C) levels, reducing triglycerides and blood glucose concentrations, and improving insulin sensitivity. Beyond these metabolic advantages, BF confers long-lasting protection against bone fractures, with effects persisting well beyond the weaning period (52-54).

Within the TLGS, the protective role of BF has been extensively examined. Findings indicate that lactation lasting up to 12 months significantly decreases the risk of MetS in women (45). Furthermore, a comparative analysis of MetS incidence between women with and without gestational diabetes mellitus (GDM) demonstrated an inverse relationship between total lactation duration and MetS risk, with a hazard ratio (HR) of 0.98 (95% CI: 0.98 - 0.99). This suggests that every additional month of BF reduces the hazard of developing MetS by 2%. Notably, among women with a history of GDM, longer durations of exclusive BF were associated with even greater reductions in MetS incidence (HR 0.93; 95% CI: 0.88 - 0.98) (55). However, given the hypoestrogenic state associated with lactation, its impact on bone health remains critical (56). A 2010 TLGS study revealed that, post-menopause, bone mineral density at key skeletal sites such as the femoral neck, Ward’s triangle, and lumbar spine was inversely correlated with total BF duration, highlighting a complex interplay between lactation duration and skeletal integrity later in life (57).

### 3.5. Trend of Oral Hormonal Contraceptive and Their Metabolic Consequences

The pattern of hormonal contraceptive use among married women of reproductive age participating in the TLGS has evolved over time (12). Specifically, the prevalence of hormonal contraceptive use demonstrated a declining trend from the first phase through to the seventh phase of the study. This shift reflects broader changes in contraceptive preferences and behaviors within this population, influenced by sociocultural factors, evolving family planning policies, and increased accessibility to a variety of contraceptive methods. Detailed analyses of TLGS data reveal that although oral contraceptive pills (OCPs) were the predominant method at the onset of the study, subsequent phases witnessed increasing adoption of alternative methods such as condoms, alongside a rise in traditional contraceptive use.

In the context of the TLGS, after adjustment for potential confounding variables, it was observed that women who used OCPs for more than 36 months exhibited significantly higher mean low-density lipoprotein cholesterol (LDL-C) levels compared to non-users. Furthermore, the odds of hypercholesterolemia were 1.5 times greater among prolonged OCP users relative to non-users (95% CI: 1.01 - 2.2). These findings underscore the potential long-term impact of extended OCP use on lipid metabolism, highlighting the importance of regular lipid profile monitoring in women using OCPs for extended durations to mitigate elevated cardiovascular risk (58).

## 4. Conclusions

Publications from the TLGS underscore the profound impact of reproductive lifespan events on long-term cardiometabolic and renal health outcomes in women. Early menarche emerged as a significant predictor of adverse metabolic conditions, including prediabetes, DM, and MetS. This association highlights the critical necessity for early risk stratification and the implementation of targeted preventive interventions among women experiencing early menarche. The elevated risk linked to early menarche is supported by robust epidemiological evidence demonstrating that girls who experience menarche at younger ages have substantially increased risks of developing DM and other metabolic disorders in adulthood, partly mediated by adult adiposity but also through direct biological pathways independent of BMI.

Furthermore, the markedly increased incidence of MetS and dysregulated glycemic profiles observed in women undergoing surgical menopause underscores the metabolic ramifications of abrupt estrogen withdrawal, emphasizing the need for vigilant monitoring and tailored management strategies for this high-risk group. Conversely, later ANM was associated with an elevated risk of CKD, suggesting that a prolonged reproductive span exerts complex physiological effects. Extended EEE may confer protective effects against hypertension, CVD, CKD, and bone fractures, illustrating the multifaceted role of estrogen in maintaining systemic health.

Additionally, lactation demonstrated a protective effect on long-term metabolic health, particularly among women with a history of GDM. This finding supports BF as a modifiable lifestyle factor capable of reducing the risk of MetS and related disorders, reinforcing its importance in postnatal care and chronic disease prevention.

Collectively, these results establish reproductive factors as pivotal determinants of women's cardiometabolic and renal health trajectories. They advocate for the integration of detailed reproductive histories into preventive care models and longitudinal risk assessment frameworks, fostering more personalized and effective healthcare strategies aimed at mitigating chronic disease risks in women across their lifespan.

## Data Availability

The dataset presented in the study is available on request from the corresponding author during submission or after publication.
